# METANNOGEN: compiling features of biochemical reactions needed for the reconstruction of metabolic networks

**DOI:** 10.1186/1752-0509-1-5

**Published:** 2007-01-09

**Authors:** Christoph Gille, Sabrina Hoffmann, Hermann-Georg Holzhütter

**Affiliations:** 1 Institute of Biochemistry Charité, Medical Faculty of the Humboldt University, 10117 Berlin, Monbijoustraße 2, Germany

## Abstract

**Background:**

One central goal of computational systems biology is the mathematical modelling of complex metabolic reaction networks. The first and most time-consuming step in the development of such models consists in the stoichiometric reconstruction of the network, i. e. compilation of all metabolites, reactions and transport processes relevant to the considered network and their assignment to the various cellular compartments. Therefore an information system is required to collect and manage data from different databases and scientific literature in order to generate a metabolic network of biochemical reactions that can be subjected to further computational analyses.

**Results:**

The computer program METANNOGEN facilitates the reconstruction of metabolic networks. It uses the well-known database of biochemical reactions KEGG of biochemical reactions as primary information source from which biochemical reactions relevant to the considered network can be selected, edited and stored in a separate, user-defined database. Reactions not contained in KEGG can be entered manually into the system. To aid the decision whether or not a reaction selected from KEGG belongs to the considered network METANNOGEN contains information of SWISSPROT and ENSEMBL and provides Web links to a number of important information sources like METACYC, BRENDA, NIST, and REACTOME. If a reaction is reported to occur in more than one cellular compartment, a corresponding number of reactions is generated each referring to one specific compartment. Transport processes of metabolites are entered like chemical reactions where reactants and products have different compartment attributes. The list of compartmentalized biochemical reactions and membrane transport processes compiled by means of METANNOGEN can be exported as an SBML file for further computational analysis. METANNOGEN is highly customizable with respect to the content of the SBML output file, additional data-fields, the graphical input form, highlighting of project specific search terms and dynamically generated Web-links.

**Conclusion:**

METANNOGEN is a flexible tool to manage information for the design of metabolic networks. The program requires Java Runtime Environment 1.4 or higher and about 100 MB of free RAM and about 200 MB of free HD space. It does not require installation and can be directly Java-webstarted from .

## Background

**Figure 1:**
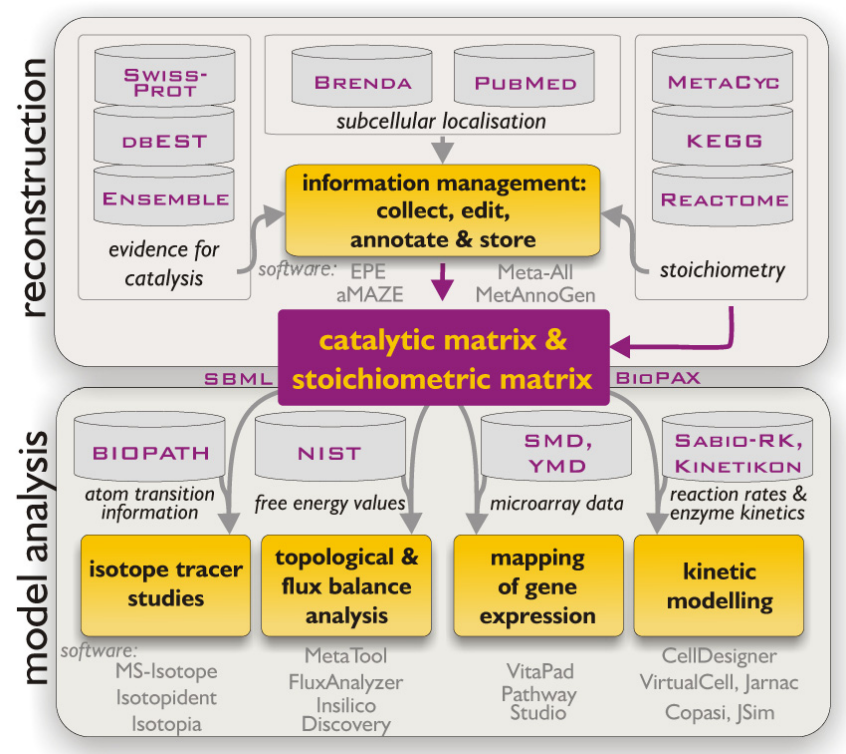
Intercorrelation of METANNOGEN with knowledge-bases and network modelling tools. The user manually collects information from biochemical reactions. The network is exported as a SBML file and may be processed with network modelling/analyzing tools.

One important goal of computational systems biology is the development of mathematical models for complex metabolic reaction networks. The type of such models and their predictive capacity depend on the available biochemical knowledge. Generally, one may distinguish two main steps in network modelling: (i) stoichiometric network reconstruction and (ii) network analysis (see figure 1). Necessary prerequisite for any type of mathematical network model is the knowledge of the network stoichiometry, i. e. reactions, membrane transport processes and associated metabolites. For eukaryotic cells, stoichiometric reconstruction of the network includes the assignment of reactions to cellular compartments. Based on the stoichiometric matrix of the network one may perform structural analyses as, for example, identification of elementary flux modes [1], possible routes for a self-consistent expansion of the network starting with some initial seed compounds [2] or calculation of stationary flux distributions based on constraint optimization [3-5]. One central issue in such analyses is to define the possible directionality of a cellular reaction. To decide on this one has to know the (Gibb's) standard free energy change of the reaction and the range within which the ligand concentrations may vary. Compared to structural modelling, a deeper insight into the regulation of reaction networks in response to external variations can be achieved on the basis of kinetic models. For the establishment of kinetic network models rate equations for all reactions and transport processes need to be set up. SABIO-RK and the Chemical Kinetics Database NIST collect kinetic data from the literature. Moreover, application of structural modelling approaches to complex eukaryotic cells requires information on the localization of the reactions in the various intracellular organelles as well as the transport of metabolites among the organelles. The biochemical information required for stoichiometric network reconstruction is spread over various data sources.

Comprehensive collections for biochemical reactions are KEGG [6], BIOCYC, [7], REACTOME [8] and UM-BBD [9]. For information on cellular compartmentalization and substrate specificity the enzyme database BRENDA [10] is valuable. Sometimes the cellular compartment of an enzyme reported in databases or publications might not be the site of its action. For example, a mitochondrial enzyme might be firmly attached to the mitochondrial wall and catalyses the biochemical reaction not inside but outside the mitochondrial matrix. Therefore literature search is necessary to obtain detailed knowledge on the biochemical reactions under consideration. There are several approaches to combine information from many sources. The AMAZE LIGHTBENCH combines a variety of information which is accessible with a web browser [11]. Available modelling programs usually allow to edit biochemical networks with a graphical network editor. This approach is sufficient for small networks but for large networks comprising many metabolic reactions an information storage system is required. The recently developed database system META-ALL allows users to enter data on biochemical reactions into a locally running ORACAL database with Web-clients [12]. The resulting model can be exported in SBML format. The program EPE is a visual editor for biological networks including metabolic networks and allows to add annotations.

To facilitate the exploitation of various information sources for the stoichiometric reconstruction of metabolic networks we have developed the interactive computer program METANNOGEN (Figure 1). In contrast to other programs for building user defined reaction network METANNOGEN uses the database KEGG of biochemical reactions as primary information source from which biochemical reactions relevant to the considered network can be selected, edited and stored. The advantages of our approach are that (I) only reaction equations not stored in KEGG need to be entered manually, (II) that the graphical pathway representation of KEGG are used, which look familiar because their layout resembles figures in text books on Biochemistry,(III) that the KEGG database can be updated without affecting the user data and (IV) that the immutable identifiers are provided for compounds and reactions by KEGG.

## Implementation

The program comes as a single executable Jar-file and does not require installation. When the Jar file is started for the first time the KEGG data files are downloaded from the KEGG-server and stored on the hard-disk. To update the KEGG database the user only needs to delete these files. The next time the program is started it will download the current version. The KEGG data is loaded into the main memory each time the program is started. This process has been highly optimized with respect to speed and memory usage to allow loading of the huge KEGG database within about five seconds on an i386 PC with a CPU tact of 2 GHz. Keeping the KEGG database in the main memory of the local PC assures extremely short access times and thus avoids the typical delays of Web interfaces set up on top of a remote database server.

**Figure 2:**
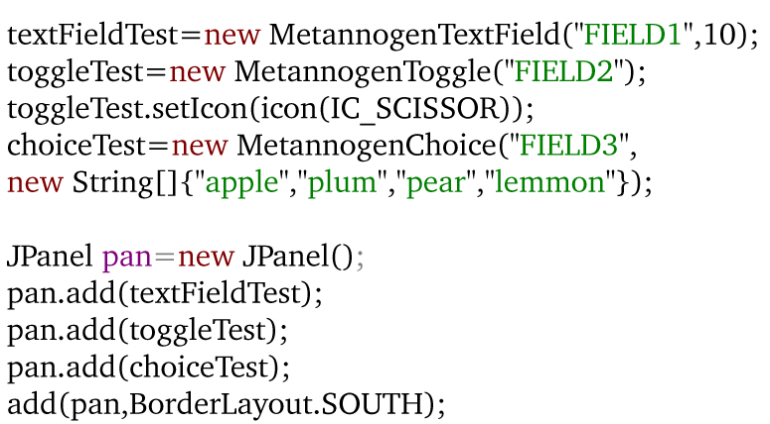
Code fragment exemplifying the extension of the graphical input form by three additional GUI elements. It exemplifies a text-field, a toggle button and a choice menu. The three components are placed in one JPanel which is added to the south.

User data and data of the KEGG database are stored in separate variables, though they might be linked if they share the same reaction ID. Therefore the program is not confined to reactions contained in KEGG. To facilitate customization of the input form and the SBML output users can modify Java source code which is instantly compiled by the embedded compiler (Figure 2).

The technique used to change and compile code at runtime has been described recently [13]. No additional software tools are required. METANNOGEN does not necessarily require a central data storage since all datasets created by the investigator are stored locally. These files could be shared with floppy-disks or other portable storage media. However, to facilitate network reconstruction in a team of investigators, a central repository may be implemented using a Web-server located in the local network. For this purpose each dataset contains the ID of the author who created the dataset.

### Limitations

• For reactions not contained in KEGG the user  needs to type the reaction  equation..

• The METACYC database is not used as a primary data source.

• Currently, METANNOGEN is not yet able to perform consistency checks on the network such as identification of unbalanced metabolites.

• Known Bugs: The graphical tree occasionally collapses. It can be rescued with the [redraw]-button.

## Results and discussion

The most comprehensive database of metabolic reactions is KEGG [14,15] which is the reason why KEGG was used as a skeleton. As a consequence reactions from other sources as for example METACYC [16] have to be entered manually. The KEGG data is represented as an expandable tree because this is an efficient form to display the complex relationships between pathways, reactions, enzymes and compounds. Reactions are child nodes of enzymes and vice versa because, eventually, one reaction may be catalyzed by several enzymes and, conversely, one enzyme may catalyze different reactions.

Reactions taken over from the KEGG database into the user-defined network carry the KEGG reaction identifier so that any alterations of reactions in the KEGG database can be easily detected and possibly transferred to the corresponding reactions in the user-defined data. For reactions not stored in KEGG the user defines an alphanumeric identifier. Transport processes are not contained in the KEGG database. They have to be entered manually using the same notation as for chemical reactions with the difference that substrates and products have different compartment attributes. The user can type compounds into the text-field of the biochemical equation using common names like "L-Citrulline", "Citrulline" or "Citrullin" and can insert compounds found on a comprehensive list of compounds. Compound names may be converted into the respective KEGG ID as for example "C00327" for L-Citrulline if the compound is contained in KEGG. All transport processes and those reactions that do not have a corresponding entry in KEGG are bundled under the tree branch "orphan" reactions or "transporters", respectively. METANNOGEN allows to specify more than one sub-cellular compartment for one single biochemical reaction which results in the generation of one dataset for each compartment. Labeling of all datasets with the user name and protection against unintended modification allows METANNOGEN to be used by a team of investigators.

### Working with METANNOGEN – a case study

**Figure 3:**
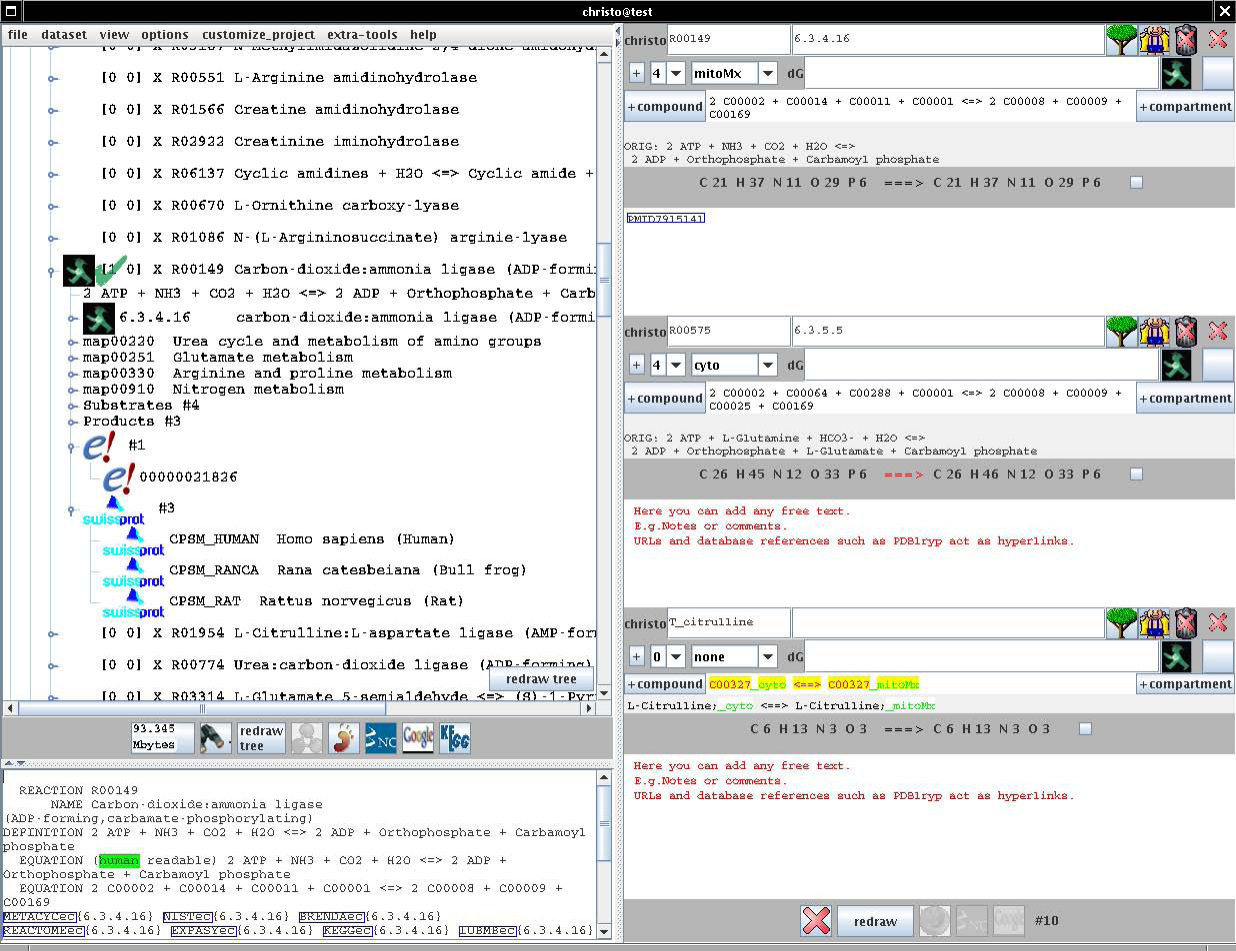
Screen-shot of the application. The upper left part shows KEGG-data as a graphical tree. The tree node of the reaction R00149 is expanded. The traffic light icon indicates that this reaction node has at least one dataset. The sub-nodes of R00149 contain information on the EC-number 6.3.4.16, the biochemical pathway maps where this reaction is contained, the four substrates and three reaction products, the ENSEMBL entry ENSG00000021826 and the three SWISSPROT entries. The lower left panel contains the textural description of the reaction. Words with a blue frame act as hyperlinks. The right part shows datasets for the two carbamoylphosphate synthetases R00149 and R00575 and the transport of citrulline across the inner mitochondrial membrane. The biochemical equation for R00149 and R00575 were taken from KEGG and the equation for the transport process was entered manually. The red arrow in R00575 indicates that there is a slight error in the equation. The left side of the equation has an hydrogen less than the right side. This can be corrected by adding " + H+" into the equation. The "H+" will automatically be replaced by "C00080". One can also use the button [compound] to select hydrogen from a list of compounds.

In the following the usage of METANNOGEN is explained using as an example the synthesis of carbamoylphosphate. The figure 3 shows the expandable tree with the tree node for Carbamoylphosphate synthesis I (CPS I) and the datasets for CPS I and CPS II as well as the citrulline transporter. The synthesis of carbamoylphosphate is the first step in the pyrimidine biosynthesis, the arginine biosynthesis, and the urea cycle. Formation of mitochondrial carbamoylphosphate used in the urea cycle is catalyzed by the enzyme carbamoylphosphate synthetase I (CPS I). The notation of the reaction in KEGG reads

2*ATP *+ *NH*_3 _+ *CO*_2 _+ *H*_2_*O *⇄ 2*ADP *+ *Orthophosphate *+ *Carbamoylphosphate *    (1)

To add this reaction to the model the respective tree node with the reaction identifier R00149 and the EC number 6.3.4.16 needs to be marked in METANNOGEN. Selecting "new dataset" from the dataset menu creates a new dataset for the carbamoylphosphate synthetase reaction in the user-defined database (Figure 3). The input mask for the dataset appears in the right side of the screen. A toggle button with the traffic light symbols allows to exclude data sets from the stoichiometric matrix without deleting the dataset. This is a useful option if a reaction and its catalyzing enzymes are not reported to exist in the species considered by the user (e.g. human hepatocyte) whereas for a related species (e.g. rat hepatocytes) such evidences are available.

Checking the consistency of the stoichiometric matrices generated with active or inactive toggle button of such "likely" reactions may provide valuable heuristics for further experimental work. For the model of the liver metabolism the reaction of carbamoylphosphate synthetase is activated since the reaction takes place in human liver without doubt. The first two reactions of urea cycle, the formation of carbamoylphosphate and the ligation with ornithine, take place in the mitochondrial matrix. This knowledge is usually obtained from databases such as Brenda [10], UM-BBD [9], SABIO-RK or from scientific articles. For convenience, some databases are linked and the respective pages are opened in the Web browser when the links are clicked.

This set of databases is customizable. For the considered reaction of the mitochondrial carbamoylphosphate synthetase the compartment "MitoMx" (mitochondrial matrix) is selected by the user in the selection box for sub-cellular compartments. The newly created dataset can hold any kind of additional information on the reaction and the catalyzing enzyme. To keep track of the source of knowledge, notes and remarks taken from literature can be entered as free text. Pubmed abstracts can be referred to simply by their ID. The abstracts are automatically downloaded and shown with important keywords highlighted. If, for example, the reference PMID:7915141 for an article on carbamoylphosphate synthetase is contained in the text-field and the mouse is moved over it, the abstract is automatically shown and important search terms like "human" or "liver" are highlighted. In general a link to a database is formed by a database ID followed by colon, in this case PMID:, and an entry ID, in this case 7915141. An alternative syntax with curly brackets allows references to terms containing characters other than digits and letters as for example BRENDAec{6.3.4.16} or GOOGLE{carbamoylphosphate mitochondrial} (Figure 3). The URLs of databases can be edited in the customize menu. In KEGG most reactions are displayed graphically in (so-called KEGG-maps). METANNOGEN can display information within these graphical pathway views. To quickly locate the reaction of the CPS I in the graphical pathway view "urea cycle"' it can be marked in the object tree and is highlighted by a red frame in the pathway view. This allows to quickly locate a reaction, such as CPS I in the context of a certain pathway. Choosing "any" in the compartment selector of the KEGG-map of a pathway, here the urea cycle, all reactions of the user-defined network are highlighted by filled colored rectangles irrespectively of their compartments. Depending on the status of the exclusion toggle mentioned before, a filled green box indicates that it is included into the model in contrast to a red box which would indicate that it is currently excluded. If a specific compartment is selected for the KEGG-map a yellow box points out that this reaction does not exist in this but in another compartment. For our example this view reveals that the compounds citrulline_mitoMx and citrulline_cyto cannot be balanced because both reactants occur in only one reaction (ornithine transcarbamylase in the mitochondrial matrix and argininosuccinate synthetase in the cytosol). In such cases the user needs to search for a possible exchange processes of the corresponding metabolite across the membrane separating the two compartments. For citrulline, a transport across the inner mitochondrial membrane is well-described in the literature.

The corresponding notation in METANNOGEN reads

*citrulline_cyto *⇄ *citrulline_mitoMx *    (2)

**Figure 4:**
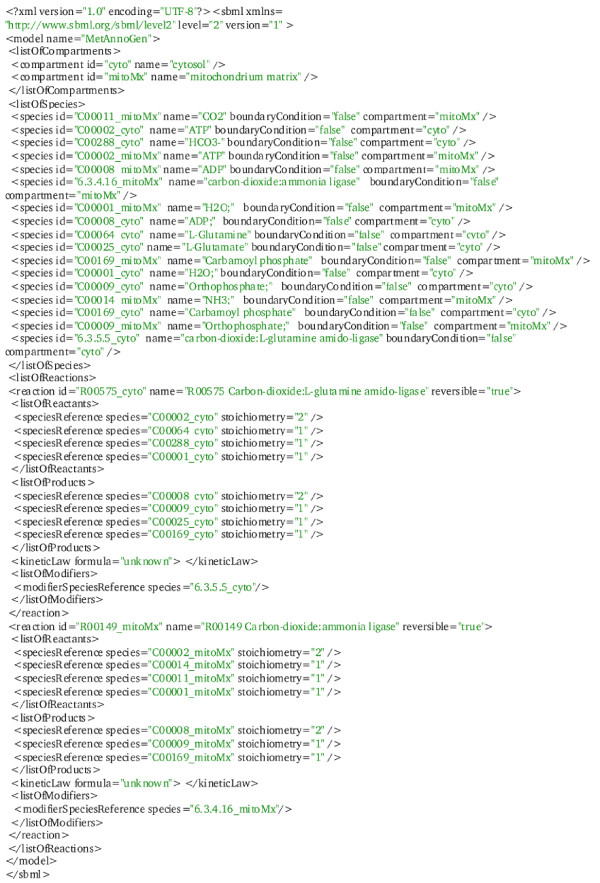
SBML-output containing the two enzymes described in section "Working with METANNOGEN – A case study". This file can be imported into modeling or simulation programs with SBML support.

To find out whether genes, transcripts and proteins have been identified for a particular enzymatic activity in men the ENSEMBL and SWISSPROT branches of the tree are helpful. They can be expanded from the reaction nodes. CPS 1 has the SWISSPROT entry CPSM_HUMAN and the ENSEMBL gene entry ENSG00000021826. A different Carbamoylphosphate synthetase CPS II catalyses the primary step of the purine synthesis. The reaction mechanism is different from that of CPS I in that the nitrogen does not originate from ammonia but from glutamine. Consequently the reaction ID and the enzyme code are different. Because the CPS II is a cytosolic enzyme "cytosol" must be selected. The SBML output for the two reactions is shown in figure 4.

### Customization of METANNOGEN for specific projects

Depending on the type of mathematical model that has to be developed for a metabolic network different types of information on the kinetics and thermodynamics of reactions and transport processes are needed. In principle, the text-area for notes can be used for such information. However, to store all information in a unified and structured manner advanced users may also create specific GUI elements such as pull-down menus toggle buttons, text-fields and check-boxes in the Java source code. For this purpose the customize menu offers the possibility to generate a copy of the GUI Java file which than replaces the default GUI Java class in the running application. The user can edit this copy. The source text contains example code for three additional GUI-elements which can be activated by removing the "/*" and "*/" tags. This renders the lines enclosed in "/* ... */" active as soon as the modified source code is saved and the additional GUI elements immediately appear. In this example the additional GUI elements correspond to data fields named "FIELD1", "FIELD2" and "FIELD3'. Meaningful names can be given instead. By default additional data fields are not exported as SBML. Nevertheless, the SBML output can be adapted using the mechanism mentioned above [13].

Again it involves direct manipulate of source code and instant testing of the modified SBML writer at runtime. This is not critical because a copy of the original file is modified by the user. Elimination of this file immediately reverts the program to the original state. In the Java code the text contents of a field can be requested by invocation of the instance method of dataset objects *String metannogenDataset#getField(String data_field_name).*

### Searches with Pubmed and Google

One major obstacle of finding relevant information using search engines is that biochemical enzymes may be named in many different ways. METANNOGEN meets this difficulty by combining synonymous names for the enzymes of interest by logical "or" to form a sensitive search query for Google or Pubmed. The searches typically result in a larger number of hits. For Pubmed abstracts additional aids are available. Moving the mouse-pointer over a Pubmed ID the abstract is downloaded into the local cache and displayed. All user defined keywords are highlighted in the abstract to quickly assess the relevance of the publication. For large numbers of publications this approach is much more efficient than opening the abstracts in the Web browser. Search queries can be entered as a hyper-references into the remark-field using the syntax PUBMED{carbamoylphosphate [TI] AND liver AND human}.

## Conclusion

METANNOGEN stream-lines the design of large metabolic networks. The metabolic network can be exported as SBML for further analysis.

## Availability and requirements

METANNOGEN is free of charge and can loaded . The software is distributed under the GNU GPL license. It loads database files from the KEGG-server. Therefore users should also check the license of the KEGG-database. The program requires Java Runtime Environment 1.4 or higher by Sun or IBM and about 100 MB of free RAM and about 200 MB of free HD space. It is tested on MS-Windows and Linux and should also run on any other platform. It does not need any advanced infrastructure like database server or Web server to operate.

## Authors' contributions

• CG has developed the program

• SH has made substantial contributions to conception and design, and program testing.

• HH has supervised the project and improved the design and tested the program.
